# The role of melatonin in polycystic ovary syndrome: A review

**DOI:** 10.18502/ijrm.v17i12.5789

**Published:** 2019-12-30

**Authors:** Sina Mojaverrostami, Narjes Asghari, Mahsa Khamisabadi, Heidar Heidari Khoei

**Affiliations:** ^1^ Department of Anatomy, School of Medicine, Tehran University of Medical Sciences, Tehran, Iran.; ^2^ Department of Molecular Medicine, Faculty of Medical Biotechnology, National Institute of Genetic Engineering and Biotechnology, Tehran, Iran.; ^3^ Faculty of Veterinary Medicine, Urmia University, Urmia, Iran.; ^4^ Student Research Committee, School of Medicine, Shahid Beheshti University of Medical Sciences, Tehran, Iran.; ^5^ Department of Biology and Anatomical Sciences, School of Medicine, Shahid Beheshti University of Medical Sciences, Tehran, Iran.

**Keywords:** Hyperandrogenism, Infertility, Melatonin, PCOS.

## Abstract

**Background:**

Polycystic ovary syndrome (PCOS) is a widespread endocrine disorder, affecting approximately 20% of women within reproductive age. It is associated with hyperandrogenism, obesity, menstrual irregularity, and anovulatory infertility. Melatonin is the main pineal gland hormone involved in the regulation of the circadian rhythm. In recent years, it has been observed that a reduction in melatonin levels of follicular fluid exists in PCOS patients. Melatonin receptors in the ovary and intra-follicular fluid adjust sex steroid secretion at different phases of ovarian follicular maturation. Moreover, melatonin is a strong antioxidant and an effective free radical scavenger, which protects ovarian follicles during follicular maturation.

**Objective:**

In this paper, we conducted a literature review and the summary of the current research on the role of melatonin in PCOS.

**Materials and Methods:**

Electronic databases including PubMed/MEDLINE, Web of Science, Scopus, and Reaxys were searched from their inception to October 2018 using the keywords “Melatonin” AND “Polycystic ovary syndrome” OR “PCOS.”

**Results:**

Based on the data included in our review, it was found that the administration of melatonin can improve the oocyte and embryo quality in PCOS patients. It may also have beneficial effects in correcting the hormonal alterations in PCOS patients.

**Conclusion:**

Since metabolic dysfunction is the major finding contributing to the initiation of PCOS, melatonin can hinder this process via its improving effects on metabolic functions.

## 1. Introduction

Polycystic ovary syndrome (PCOS) is a complex disorder arising from the interaction of genetic and environmental reasons that affects up to 20% of women at reproductive age (1). According to the ESHRE/ASRM consensus, at least two of the following three features should be present for proper PCOS diagnosis: 1) ovulatory dysfunction (oligoanovulation and/or anovulation); 2) hyperandrogenemia (the biochemical feature of androgen excess) or hyperandrogenism (the clinical feature of androgen excess); 3) polycystic appearance of ovaries in ultrasonography (2), together with the exclusion of other etiologies (3). In addition to reproductive and cosmetic sequelae, PCOS syndrome is associated with a higher risk of metabolic disorders including insulin resistance, increased oxidative stress (4), cardiovascular disease, type 2 diabetes mellitus, liver disease, and endometrial cancer (5, 6).

Women with PCOS often seek treatment due to their complaints of infertility and menstrual cycle irregularities which are the results of chronic oligo/anovulation (7). Changing lifestyles, such as nutritional counseling and weight loss are the necessary step of all treatment plans (8). Despite the many advances in the understanding of the Pathobiology and treatment strategies of PCOS over the past decades, many questions remain to be answered and the treatment of the syndrome remains empirical.

Melatonin (N-acetyl-5-methoxytrypamine) is an indolamine hormone that was firstly recognized in the 1950s (9). Melatonin levels are regulated by photoperiod as its production and secretion are promoted at night in response to darkness since light can suppress its secretion (9). Melatonin is also produced in other organs such as the gastrointestinal tract, skin, retina, bone marrow, and lymphocytes (10, 11). It seems that mitochondria are the site of melatonin synthesis within cells. Moreover, the female reproductive organ, including the follicular cells, oocytes, and cytotrophoblasts are also among the melatonin production sites (12). Several studies have shown the involvement of melatonin in the pathogenesis of diabetes, cancer, Alzheimer's disease, immune and cardiac diseases (13-15). Melatonin has been identified to have different pharmacological properties such as antioxidant, immunomodulatory, anti-angiogenic, and oncostatic effects (16). Melatonin acts as an inhibitory factor on the hypothalamic pituitary gonadal axis (17). Melatonin receptors are transmembrane G-protein-coupled receptors including melatonin receptor 1 (MT1; *MTNR1A*) and melatonin receptor 2 (MT2; *MTNR1B*) (18).

The effects of melatonin on female reproductive physiology are mediated via its receptors in hypothalamic, pituitary, and ovarian sites (19). Melatonin is also a potent free radical scavenger that exerts protective effects in female reproductive organs; for instance, it is involved in the protection of the oocyte against oxidative stress, particularly at the time of ovulation. It can also be used to protect the developing fetus from oxidative stress can be happened by melatonin (20). The levels of melatonin in follicular fluid are higher than its levels in the blood (21). The concentration of melatonin in follicular fluid significantly rises as the follicles become mature (22).

In this paper, we have made an effort to review the possible roles of melatonin in the pathogenesis of PCOS as well as the potential melatonin-centered therapeutic measures, which can be recruited herein.

## 2. Materials and Methods 

### Search strategy

Related published articles were searched in the following electronic databases: PubMed/MEDLINE, Web of Science, Scopus and Reaxys. All the sources were searched from their inception to October 2018 using the keywords “Melatonin” AND “Polycystic ovary syndrome” OR “PCOS.”

### Study selection

**Figure 1:**
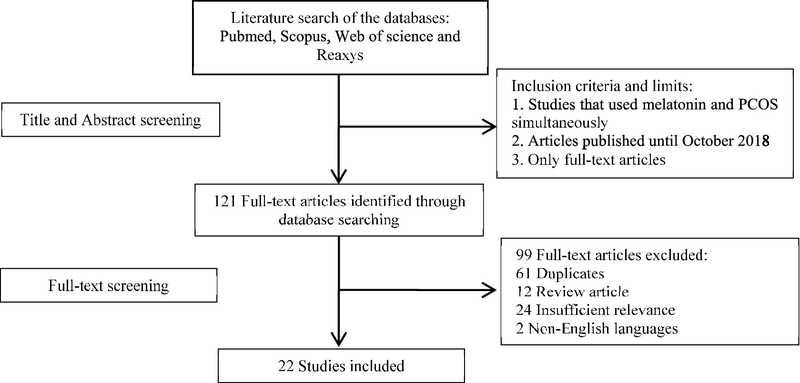
Flow diagram of the search strategy.

Titles and abstracts from the electronic databases were scrutinized following the search for the keywords, and the full-text papers which were expected to match with our inclusion criteria were obtained. The following specified inclusion criteria were used: (1) studies that used melatonin in context of PCOS or those in which melatonin was somehow shown to be related to any of the mechanisms involved in the pathogenesis of PCOS; (2) papers with available full texts; (3) Papers in English language only. There was no limitation regarding the inclusion of any human or animal models for PCOS or cell lines researches. Furthermore, the following exclusion criteria were applied: (1) no report on the treatment with melatonin; (2) no report with the PCOS disease; (3) no report on the use of melatonin for treating PCOS in clinical or animal models or cell lines researches; (4) no report on the melatonin level alternation in the different body biological fluids; (5) review articles; and (6) non-English language articles (Figure 1).

### Data extraction

The papers, meeting all the aforementioned criteria were reviewed separately to confirm the reliability of the extracted data. All characteristics of the articles such as author name, year, study location, study population, study period, treatment method, dose of treatment, and outcomes were extracted from the papers. The extracted data of the selected studies are shown in Tables I and II.

**Table 1:** Changes in Melatonin levels of PCOS patients


**Author, year (ref)**	**Country**	**Participants**	**Mean age (year)**	**Sample**	<**Melatonin or its metabolite levels α MT6s in urine (µg/24 h) Melatonin in saliva, blood, and follicular fluid (pg/ml)**		<**Hormonal levels in serum LH, FSH (mIU/ml) Testosterone(ng/ml) 8-OHdG (ng/ml)**			**Result**
			Patients	Control	LH/FSH ratio	Patients	Control	
Luboshitzky *et al*., 2003 and 2004 (33, 34)	Israel	12 women with PCOS	20.3 ± 4.8	Urine	αMT6s: 52.6 ± 20.3	30.5 ± 6.5	LH FSH T	13.5 ± 2.9 5.3 ± 2.1 0.58 ± 0.28	4.6 ± 2.2 6.5 ± 1.4 0.3 ± 0.1	The level of LH, testosterone, and αMT6s were significantly higher in PCOS patients
Luboshitzky *et al*., 2001 (35)	Israel	22 women with PCOS	22.9 ± 5.2	Urine	αMT6s: 54.0 ± 20.3	30.1 ± 6.6	LH/FSH	2.04 ± 1.24	0.74 ± 0.39	PCOS patients had higher levels of aMT6s, testosterone, LH/FSH ratio, and insulin than control women. Testosterone was a good indicator for aMT6s concentration in PCOS patients which inversely related to aMT6s
Shreeve *et al*., 2013 (42)	UK	15 women with PCOS	29.8+3.7	Urine	αMT6s: 60.3 ± 30.6	37.7 ± 21.5	FSH LH T 8-OHdG	6.9 ± 0.6 8.1 ± 1.2 2.3 ± 0.2 120.5 ± 42.1	84.0 ± 40.8	Night-time melatonin and 8-OHdG concentrations were significantly higher in PCOS women compared to healthy women
Jain *et al.*, 2013 (40)	India	50 women with PCOS	24.87 ± 4.43	Blood plasma	Melatonin: 63.27 ± 10.9	32.51 ± 7.5	FSH LH T LH/FSH	5.43 ± 1.53 16.13 ± 7.95 1.2.84 ± 6.96 3.10 ± 1.69	6.60 ± 2.86 7.18 ± 1.97 0.84 ± 12.75 1.15 ± 0.25	Melatonin level increase in all the cases of PCOS women but testosterone level rise in 72% of patients. Melatonin level positively related to increased testosterone concentration
Terzieva *et al*.**,** 2013 (49)	Bulgaria	30 women with PCOS	25.07 ± 1.10	Blood serum	Melatonin: 49.37 ± 3.79	42.91 ± 9.38	FSH LH T LH/FSH	6.83 ± 1.04 12.43 ± 3.83 0.60 ± 0.05 1.64 ± 0.33	5.57 ± 0.32 4.08 ± 0.35 0.32 ± 0.03 0.73 ± 0.03	In PCOS women, serum melatonin concentration was significantly higher than the healthy women
Zangeneh *et al*., 2014 (50)	Iran	77 women with PCOS	26.6 ± 4.7	Blood serum	Melatonin: 25.48 ± 24.27	32.45 ± 24.27	–	–	–	Melatonin concentration in serum of PCOS women was significantly lower than the control women
Kim *et al*., 2013 (72)	South Korea	13 women with PCOS	31.1 ± 0.8	FF	Melatonin: 20.9 ± 3.6	136.8 ± 26.1	–	–	–	In women with PCOS, melatonin concentration in follicular fluid was significantly lower than the control group

**Table 2:** Protective effects of exogenous Melatonin in PCOS


**Author, year (ref)**	**Country**	**Study design**	**PCOS model**	**Sample size**	**Treatment (dosage)**	**Route of administration**	**Duration**	**Results**
Lemos *et al*., 2016 (68)	Brazil	In-vivo	PCOS rats (induced by constant light stimulation)	50	Combination of melatonin (200 μg/100 g b.w.) and metformin hydrochloride (50 mg/100 g b.w.)	Melatonin (subcutaneous injections) and Metformin hydrochloride (gavage)	20 days	Reduced the time needed for pregnancy and reduced plasma estrogen levels in the treated group, increased the plasma progesterone levels and the number and weight of offspring, besides improving fetal development
Kim *et al*., 2013 (72)	South Korea	In-vitro	PCOS women	13	IVM medium containing 800 µl IVM medium with 10 µmol /l melatonin	–	2 days	Addition of melatonin leads to improve in the cytoplasmic maturation of immature oocytes and also implantation rates and pregnancy rates were enhanced
Pacchiarotti *et al*., 2015 (64)	Italy	In-vivo	PCOS women	165	Combination of myo-inositol (4000 mg), folic acid (400 mcg) and melatonin (3 mg)	Orally administered	14 days	Increasing the number of mature oocytes in the treated group was showed and intra-follicular concentration of melatonin was four times higher than in the control group
Lemos *et al*., (2014) (83)	Brazil	In-vivo	PCOS rats (induced with constant Illumination)	15	Combination of melatonin (200 μg/100 g b.w.) and metformin hydrochloride (50 mg/100 g b.w.)	Melatonin (subcutaneous injections) and Metformin hydrochloride (gavage)	20 days	Combination of two drugs was more helpful in the decline of plasmatic levels of liver enzyme, nitric oxide, and total glutathione. Also, in the treated group, inflammatory response and histopathological damage were decreased
Lima *et al*., 2004 (79)	Brazil	In-vivo	PCOS rats (Induced with pinealectomy or continuous light)	113	Melatonin (200 μg/100 g body weight)	Injected intramuscularly	4 months	Melatonin-treated groups showed a significant reduction in the number of cysts and antigonadotrophic effects
Nikmard *et al*., 2016 (73)	Iran	In-vivo/In-vitro	PCOS mice (induced by the injection of dehydroepiandrosterone)	16	10^-5^, 10^-6^, and 10^-7^ mol/L of melatonin were added into the medium culture.	–	24 hours	Addition of melatonin to medium culture increased maturation rate and cleavage rate. Highest maturation rate was observed at 10^-6^ mol/L concentration of melatonin
Pai *et al*., 2014 (56)	India	In-vivo	PCOS rats (induced by administration of testosterone)	16	Melatonin (1 mg/kg and 2 mg/kg )	Intraperitoneally injected	35 days	Both doses of melatonin meaningfully reduced the number of cystic follicles, neoplastic endometrial glands and decreased adipocyte hypertrophy
Ahmadi *et al*., 2017 (74)	Iran	In-vivo	PCOS mice (induced by injections of DHEA)	12	Melatonin (10 mg/kg body weight)	Intraperitoneally injected	5 days	Administration of melatonin leads to a significant increase in the thickness of the granulosa layer but the reduction in the thickness of the theca layer
Tagliaferri *et al*., 2017 (91)	Italy	In-vivo	PCOS women	40	Melatonin (Armonia Fast 1 mg; 2 tablets a day)	Orally administrated	6 months	Melatonin treatment decreased androgens levels, but FSH level significantly raised and anti-Mullerian hormone level significantly dropped
Basheer *et al*., 2018 (80)	India	In-vivo	PCOS rats (induced by Letrozole)	–	Melatonin (200 μg/100 g body weight)	Intraperitoneally injected	–	Melatonin treatment in PCOS rats restored the MT1 and MT2 receptors in the ovarian tissue
Al-Qadhi, 2018 (60)	Iraq	In-vivo	PCOS women	50	Melatonin 3 mg/day	Orally administrated	2 months	Melatonin treatment decreased LH level and BMI in PCOS patients

## 3. Results 

### Study characteristics

After searching the aforementioned databases with considered keywords, the papers were reviewed by two authors. In the first step, titles and abstracts were screened and 121 papers that had enough connectivity with our keywords were included. In the second step, full texts were reviewed for the eligibility and relevance of their findings, and 99 articles were excluded due to duplicate data, non-English languages, review articles, and insufficient relevance. Finally, 22 studies were selected, including studies that have used melatonin administration for treating PCOS in clinical and animal models of PCOS and also the studies that have shown alternation of melatonin levels involved in the pathogenesis and diagnosis of PCOS; 22 articles were included in the current review, including 15 clinical human studies and 7 animal studies. Table I is related to the studies that indicate the alternation of melatonin levels in different biological fluids such as blood, serum, urine, saliva, and follicular fluid in PCOS patients. Table II is related to the studies that have used exogenous melatonin as a drug for curing PCOS in sick women, animal models, or cell lines researches. And, the remaining studies are related to the melatonin receptor gene polymorphisms in PCOS patients.

### PCOS pathogenesis

**Figure 2:**
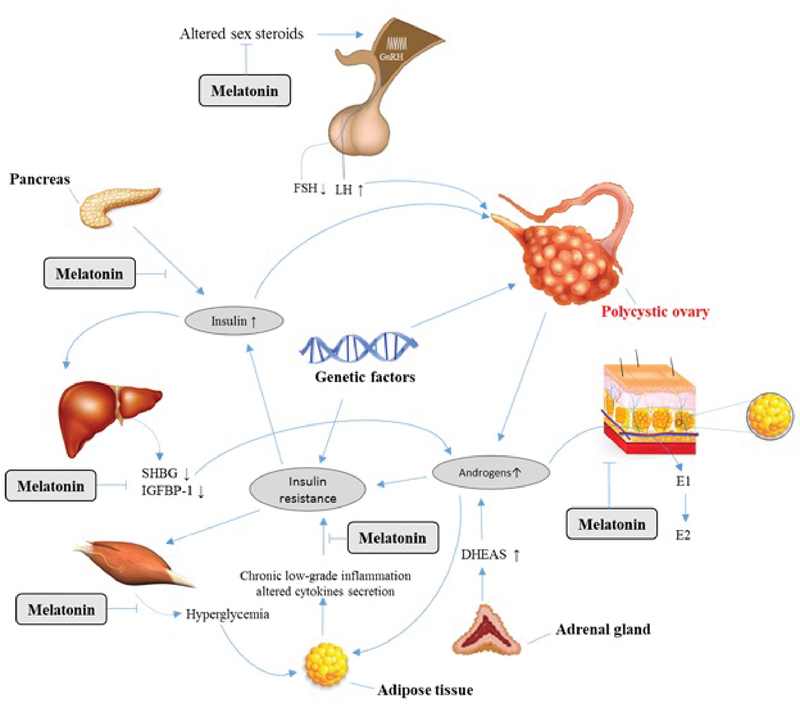
The role of melatonin in the pathogenesis of PCOS.

PCOS may be initiated by some specific abnormalities in the hypothalamus-pituitary compartment, ovaries, adrenal gland, and the peripheral compartment like adipose tissue (23). In PCOS patients, an increase in gonadotropin-releasing hormone (GnRH) pulse frequency enhances the luteinizing hormone (LH) pulse frequency and amplitude (24). In spite of elevated LH secretion, follicle-stimulating hormone (FSH) levels remain in the lower follicular range due to the negative feedback of enhanced estrogen levels and follicular inhibition (24). As a result of altered LH:FSH ratio in PCOS women, the androgen production by the theca cells in the ovaries is increased; however, due to the low FSH levels, the follicular maturation is dramatically impaired (25). In addition, ovarian dysregulation of cytochrome-17, defects in the aromatase activity of the ovarian granulosa cells (GC), as well as the stimulation of the ovarian theca cells by high levels of insulin-like growth factor 1 (IGF-1) are among other mechanisms involved in androgen overproduction (26). Excessive adrenocortical secretion of dehydroepiandrosterone is observed in approximately 50-70% of PCOS patients (27). Increased peripheral 5 alpha-reductase activity and enhanced peripheral aromatization of androgens to estrogens that induces the reversal of estrone to estradiol ratio and a chronic hyperestrogenic state are also seen in PCOS patients (28). There is general agreement that insulin resistance and hyperinsulinemia play a major part in the pathogenesis of PCOS (29). Hyperinsulinemia stimulates ovarian theca cells and leads to androgen overproduction. Hyperinsulinemia along with hyperandrogenemia and enhanced levels of IGF-1 inhibit sex hormone-binding globulin secretion, which surges the levels of bioactive androgens and worsens the clinical manifestations of androgen excess in PCOS patients (29). Also, it has been discovered that insulin resistance and hyperandrogenism can affect normal function of adipocytes (30). Previous researchers have found that adipose tissue dysfunction are associated to metabolic and reproductive dysfunction including insulin resistance and androgen excess secretion in most PCOS patients (31) (Figure 2).

### Alternation of melatonin levels in different body fluids of PCOS patients

Melatonin synthetic enzymes such as arylalkylamine N-acetyl-transferase and hydroxyindole-O-methyltransferase have been identified in most tissues including ovaries (10). Measurable levels of melatonin have been identified in most biological fluids, interestingly in the follicular fluid. The concentration of melatonin in preovulatory follicles is higher than that of plasma suggesting its possible direct effects on ovarian function (32).

In several studies, melatonin levels in PCOS women have been measured to find its role in the PCOS pathogenesis (Table I) (33, 34). The most important metabolite of melatonin, urinary 6-sulfatoxymelatonin (aMT6s), is considered to be a good marker for melatonin production in the body (35). The plasma concentrations of aMT6s in hyperandrogenic and PCOS women were significantly higher than healthy women possibly due to higher melatonin production (35). It has been demonstrated that testosterone and estrogen can regulate melatonin secretion. Melatonin secretion has been shown to decrease in castrated rats due to the reduction of testosterone levels (36). Ovariectomized rats exposed to 17β-estradiol had reduced numbers of α/β-adrenoreceptors, responsible for the stimulation of melatonin production, in their pinealocytes (37). Sex hormones can adjust the human biological clock by affecting the hypothalamic suprachiasmatic nucleus and pinealocytes (38). In a study by Luboshitzky and colleagues, PCOS women had higher levels of αMT6s, LH, and testosterone than patients with idiopathic hirsutism or the control groups. The results of this study showed that αMT6s level inversely related to testosterone level in PCOS disease. However, this result seems contradictory because other studies in PCOS introduced the direct relationship between αMT6s and testosterone (33). Treatment with estradiol-cyproterone acetate normalized the αMT6s levels in PCOS patients by inhibiting androgens and gonadotropins (33). In another study by the same group, women with PCOS had higher aMT6s, testosterone, LH/FSH ratio, and insulin values than women in the control groups. Higher aMT6s levels were due to the amplification of melatonin production. In some studies, testosterone was introduced as a determinant of aMT6s level in PCOS patients (35). Melatonin has been found to increase the secretion of progesterone and androgen in pre-antral follicles after incubation for two weeks and in antral follicles after a 30-hr incubation (39). In other studies, the elevated melatonin levels in serum of PCOS patients was found to be positively associated with testosterone levels (40). Results from studies show that melatonin levels in blood and saliva along with the level of 6-sulfatoxymelatonin in urine were significantly higher in PCOS patients compared to the healthy women (41). The levels of 6-sulfatoximelatonin in urine, nocturnal melatonin levels in saliva (at 3:00 am), and melatonin in the blood had a significant correlation with the degree of sleep disorders (41). 8-hydroxy-2'-deoxyguanosine (8-OHdG) is a marker of oxidative damage to DNA that can be detected in urine (42). In one study, the daytime urinary aMT6s and 8-OHdG levels were similar in PCOS patients and the control group, while the night-time levels of these molecules were significantly higher in PCOS patients than those of the control group (42). During the night, PCOS women with raised oxidative stress markers had higher levels of 8-OHdG and aMT6s. The production of higher amounts of melatonin was probably for neutralizing reactive oxygen species (ROS) at the night time. Melatonin level in PCOS cases was shown to have a significant correlation with the serum LH:FSH ratio (40). In patients with higher LH:FSH ratios, melatonin levels were significantly lower than the patients with lower ratios, indicating the inverse correlation of LH:FSH ratio and melatonin secretion (40).

Different studies have indicated that PCOS patients have higher serum levels of melatonin (35, 40), therefore melatonin could be used as a valuable marker for the prediction of PCOS. It was suggested that the elevation of serum melatonin in PCOS patients is due to the reduction in its follicular fluid concentration (32) The role of melatonin in oocyte maturation has been also approved. Concentration of melatonin in the preovulatory follicles is higher than smaller immature follicles, resulting in higher antioxidant capacity of larger follicles (43). In PCOS patients, the decline in the follicular concentration of melatonin is due to the reduction in the uptake of melatonin from circulation and an increase in the numbers of atretic follicles (32). Follicular atresia could be seen in PCOS patients due to increased oxidative stress and follicular damage which occurs as a result of the reduction in intra-follicular melatonin levels (32). A great deal of research has shown that ROS generation and lipid peroxidation are meaningfully higher in PCOS cases (44, 45). Along with these changes, levels of superoxide dismutase, catalase, and glutathione peroxidase are reduced, which causes intense oxidative stress in ovarian follicles (46). Melatonin is shown to be capable of regulating the gene expression of antioxidant enzymes, in addition to preventing apoptosis by increasing Bcl2 and reducing Caspase 3 (47, 48). Terzieva and colleagues reported that melatonin levels in women with PCOS in the morning were significantly higher than at night time, and the night-day difference of melatonin levels in PCOS cases was lower than that of the healthy group (49). However, a study reported conflicting results stating that the total serum melatonin levels were significantly lower in women with PCOS (50).

### Association of melatonin receptor gene polymorphisms in PCOS patients

The positive effects of melatonin on different tissues of the body are mediated by the melatonin receptors 1A and 1B (18). The earliest study on the association between common genetic variations of melatonin receptors and the prevalence of PCOS was conducted by Wang and colleagues (51). In this study, four single nucleotide polymorphisms (SNPs) (rs4753426, rs10830963, rs1562444, and rs1279265) in *MTNR1B* gene were determined and no differences in genotype and allelotype frequencies for these SNPs were found neither in the PCOS nor in the healthy women. In addition, they found a significant association between the rs10830963 SNP and the concentration of testosterone in PCOS patients. The amount of testosterone, glucose, and insulin in the serums of women with cytosine/guanine allele (CG) and guanine/guanine allele (GG) genotypes were considerably higher than the cytosine/cytosine allele (CC) genotypes, which describes the possible effects of DNA sequence variations of melatonin receptor genes on the occurrence of PCOS. In subsequent research that was performed on Chinese women, the relationship between the pathogenesis of PCOS and rs2119882 SNP, which is located in the *MTNR1A* gene was evaluated. Genotype and allele frequencies of rs2119882 were significantly different between the PCOS cases and the controls; the C allele frequency in the PCOS patients was significantly higher than the control group which confirms the association of SNP rs2119882 with PCOS (52). In another study, an association of two SNPs, rs10830963 and rs10830962 located at the *MTNR1B* gene with PCOS, was examined. Both genotypes and allelotypes occurrences of the rs10830963 SNP in PCOS women were significantly different compared to healthy women. In addition, the occurrences of GG and GC were higher among PCOS women. According to the results of the mentioned study, rs10830963SNP is associated with the predisposition of women to PCOS. No suggestive differences were observed in the genotypes and allele distributions of the other SNPs (rs10830962) between the PCOS and the healthy women (53). Recently, Song and colleagues reported that there is a significant association between rs2119882 and the prevalence of PCOS, although no association was found between rs10830963 and PCOS (54). They reported that the clinical and metabolic features of PCOS manifest largely in CC genotype carriers than the TC and TT genotypes. They also showed that there exists a considerable difference in the transmission of allele C of rs2119882 between obese and non-obese PCOS patients.

### Protective effects of exogenous melatonin administration in PCOS

#### Metabolic function improvement by melatonin

An improvement of metabolic function in PCOS patients may enhance ovarian function. There has been growing evidence suggesting the relation of melatonin with glucose homeostasis and insulin secretion. Peschke and colleagues have shown a negative correlation between melatonin and insulin levels in patients with type 2 diabetes (55). Furthermore, several studies have shown that melatonin administration can improve glucose hemostasis, exert antihyperglycemic effects, and improve endothelial vascular function in experimental models of metabolic syndrome and type 2 diabetes (56). Faria and colleagues showed that melatonin, through melatonin receptors 1 and 2, activates a brain-liver communication and suppresses hepatic gluconeogenesis via peripheral muscarinic receptors in rats (57). Evidence from in-vitro studies have shown that glucose uptake in adipocytes and skeletal muscle cells can be increased by melatonin (58).

Letrozole is an aromatase inhibitor which is largely used for treatment of breast cancer (34). In 2001, Letrozole, for the first time, was described as an ovulation-inducting agent (35). Letrozole inhibits androgens-to-estrogens conversion at the GC, resulting in the reduction of estrogen levels, which consequently releases the hypothalamus-pituitary axis from its negative feedback and increases the FSH secretion by pituitary stimulation (35). In addition, an inhibition of aromatase activity at the ovarian level increases intraovarian androgens that improve follicular sensitivity (36). A meta-analysis including 26 randomized controlled trials (5,560 women) concluded that letrozole therapy appears to improve live birth and pregnancy rates in anovulatory PCOS patients compared with clomiphene citrate (37, 38). Chottanapund and colleagues (59) have evaluated the aromatase suppressive effects of melatonin on hormonal positive breast cancer cells and have shown that melatonin was as potent as letrozole in inhibiting aromatization of androgen to estrogen. Furthermore, in several studies, it has been demonstrated that melatonin behaves as a selective estrogen enzyme modulator (SEEM) and a selective estrogen receptor modulator (SERM) (40). Moreover, the aromatase-suppressive effect of melatonin has been also shown in various cells, such as breast cancer, glioma, and endothelial cells (42). Several studies have demonstrated that melatonin reduces obesity and restores adipokine patterns and ameliorates the proinflammatory state, which underlies the development of insulin resistance (60). These findings all together show that the use of melatonin due to its aromatase-modulating activity, as well as its reducing effects on hepatic gluconeogenesis, ameliorating the pro-inflammatory state present in PCOS, improvement of glucose uptake by peripheral tissues, and the subsequent reduction in insulin levels, may be effective in the management of PCOS patients (Table II).

### Melatonin in PCOS patients undergoing assisted reproductive technology (ART) treatment

PCOS affects ART outcomes and controlled ovarian hyperstimulation (COH) with conventional protocols leads to a higher risk of ovarian hyperstimulation syndrome (OHSS) (61). Several studies have demonstrated that the poor fertilization, low oocyte, and embryo quality adversely influence the clinical outcomes in PCOS patients undergoing ART treatment (62). Insulin resistance of ovarian GC and overexpression of vascular endothelial growth factor (VEGF) due to insulin stimulation are proposed to be the underlying mechanisms of poor clinical outcomes (63). Melatonin may affect ovarian microenvironment by improving insulin resistance. In a randomized double-blind trial of PCOS patients, the administration of melatonin with myo-inositol enhanced the oocyte and embryo quality (64). The main goal in COH of PCOS patients is to prevent OHSS. The only way to prevent all type of OHSS in ART treatment is in-vitro maturation (IVM) followed by in-vitro fertilization (IVF) since this approach avoids the activation of VEGF-mediated processes (65). However, despite the extensive research conducted so far, IVM is still a suboptimal procedure and the application of IVM in infertility treatment remains immature (66). The IVM involves additional procedures that increase oxidative stress in oocytes and embryos and reduce the developmental competence of oocytes, in comparison to the conventional IVF (67). The effects of melatonin on oocyte and embryo quality and ART outcomes are discussed in the following sections of this review.

### Fertility and pregnancy

Recently, several studies evaluated the effects of melatonin in the treatment of PCOS to improve fertility and hormonal alterations. Lemos and colleagues have shown that rat models of PCOS have lower weight gain during pregnancy and show reduced numbers of implantation sites which result in a decreased number of offspring. Also, rat models of PCOS have higher collagen content in the uterine horns which hinders the blastocyst-endometrial interactions and reduces the implantation rate (68). Co-treatment of melatonin and metformin can reverse the reduced weight gain and the high collagen fiber content of uterine horns in the PCOS group, compared to the control group. Moreover, the number and weights of the offspring, the blastocyst-endometrium interactions, and the fetal development were increased while the time required for pregnancy was decreased (68). In some recent studies, it has been reported that oral administration of melatonin has no significant effects on clinical pregnancy rate or oocyte and embryo qualities (69).

### Oocyte maturation

Follicular fluid melatonin produced by the luteinizing GC in the late folliculogenesis period has a substantial function in the growth and maturation of mammalian oocytes (47). In the ovary, melatonin receptors in granulosa-lutein cells can regulate the function of this organ by controlling progesterone secretion in addition to LH and GnRH receptors gene expression via pathways such as mitogen-activated protein kinase and activation of Elk-1 (22). Melatonin concentrations are significantly higher in larger follicles, particularly in preovulatory follicles, due to participation in the secretion of progesterone and maturation of oocyte along with subsequent ovulation and luteinization events (70). Interestingly, intrafollicular melatonin level is considerably lower in PCOS women than the healthy women which may be the reason for the anovulation and low oocyte quality (32).

The supplementation of culture medium with different substances can increase oocyte maturation and IVF, as a remedy for reproductive problems (71). Based on these findings, a number of studies were designed, using supplementation of culture media by melatonin to enhance oocyte maturation and embryonic development. In a study by Kim and colleagues, melatonin concentration gradually increased in the culture media of GC due to its production by GC. Also, the addition of melatonin to IVM media improved the cytoplasmic maturation of immature oocytes and increased the rates of implantation (72). The amplification of mRNA expression of the enzymes that participate in melatonin production in cultured GC including acetylserotonin O-methyltransferase (ASMT) and aralkylamine N-acetyltransferase (AANAT) has also been shown. PCOS results in anovulation and is considered an important cause of IVF failure. Melatonin along with other substances such as myo-inositol was identified as a good predictor for IVF outcomes, and their high concentrations were an indicator of appropriate quality of oocytes (32). Oral administration of melatonin in combination with myo-inositol in PCOS patients enhanced the quality of the oocyte and embryo, increased the number of mature oocytes, and finally resulted in an increased concentration of follicular fluid melatonin (64). In another study, the beneficial effect of melatonin on the quality of oocytes in PCOS patients during IVM with different melatonin concentrations was investigated. Also, melatonin proved to be effective in the stimulation of nuclear maturation of oocytes as well as in increasing the cleavage rate via regulating free radicals to a certain level required for increasing maturation rate (73). This effect of melatonin on oocyte maturation had a dose-dependent pattern in a manner that lower doses were more effective on maturation rate.

### Histological changes in the ovary

In one study, the histopathological inspection of the ovary and uterus showed reductions in neoplastic endometrial glands and cystic follicles in PCOS rats following melatonin treatment (56). In another study, the administration of melatonin reduced cystic follicles and thickness of the theca layer and increased the number of corpus luteum and the granulosa layer thickness (74). The reduction in the thickness of theca interna is due to the action of melatonin on the reduction of androgen production in the ovary (75). Melatonin showed protective effects on corpus luteum against ROS via its antioxidant effects (76). The absence of melatonin in pinealectomized animals causes the development of ovarian cysts due to the modification of the synthesis of LH and FSH. Increase in the LH levels is a major abnormality detected in PCOS (77). Similarly, constant illumination, an induction model of PCOS in rats, have shown permanent estrous condition and polycystic ovaries aspect (78). In one study, pinealectomy and continuous light were used separately in female rats to induce PCOS. In both of these methods, the production of melatonin was diminished and PCOS condition was observed. In the group treated with melatonin, a significant reduction in the number and size of cysts were observed, probably as a result of the antigonadotrophic effects of melatonin (79). Melatonin has shown protective effects against metabolic and reproductive abnormalities in animal models of PCOS (56, 80). Melatonin treatment in PCOS patients significantly affects body characteristics including reduced body weight, body mass index, and intra-abdominal fat (56).

### Antioxidant effects

Several studies revealed that oxidative stress is one of the main reasons for female reproductive system disorders such as infertility, endometriosis, and PCOS (81). This has been supported by the fact that PCOS patients have higher levels of oxidative stress compared to healthy women (45, 82). It has been demonstrated that PCOS patients are at risk of cardiovascular diseases due to their higher exposure to oxidative stress and the following undesirable effects such as blood pressure and insulin resistance (45). Lemos and colleagues showed that animal models of PCOS induced by constant illumination had higher levels of lipid peroxidation, which leads to increases in oxidative stress, pro-oxidant enzymes, and pro-inflammatory cytokines (83). Treatment with a combination of metformin hydrochloride and melatonin was shown to be advantageous in the PCOS group by regulating plasmatic variables of oxidative stress, for example, reduction of nitric oxide and total glutathione levels. Furthermore, treatment with melatonin apparently reduced liver toxicity, pro-inflammatory cytokines, TNF-α and IL-1 and the pro-inflammatory enzymes iNOS (83). Similarly, in different studies, the antioxidant effect of melatonin along with its inhibitory effects on pro-oxidant enzymes and pro-inflammatory cytokines were reported (84).

Melatonin supplementation to the culture medium has been shown to increase the oocyte maturation rate and reduce ROS production (85). It can promote the expression of superoxide dismutase and glutathione peroxidase (86); also, melatonin is able to quench ROS and reactive nitrogen species (RNS) (76). Scavenging action of melatonin plays a valuable role during ovulation, because ovulation is an inflammatory process, and reactive species are generated and released in the follicular fluid (87). Therefore, melatonin quenches ROS and RNS and protects GC and oocyte during ovulation (32). Studies have also displayed that oxidative stress is detrimental to oocyte maturation, since the activation of oxidative stress pathway in follicular and IVM medium is unavoidable due to cellular metabolism and lack of antioxidant mechanisms, melatonin is a suitable candidate to be added to the IVM medium (73, 88).

### Melatonin and menstrual cyclicity in PCOS 

Menstrual cycle irregularities are among the major complications of PCOS that affect the quality of life of patients leading to infertility (89). The altered steroid sex hormones in PCOS patients affect the hypothalamic-pituitary-ovarian axis and lead to the failure of follicular maturation and ovulation (89). Furthermore, hyperinsulinemia and insulin resistance cause ovarian dysfunction (90). These events result in anovulation and inadequate hormonal levels leading to irregularities in menstrual cycle.

Melatonin seems to promote follicular maturation and ovulation through the protection of follicles against oxidative stress and their rescue form atresia (40). After six months of melatonin therapy in 40 normal-weight PCOS patients, menstrual irregularities and hyperandrogenism were improved. The lack of significant alterations in the secretion of insulin and insulin sensitivity suggests that melatonin may act on the ovary through an independent mechanism (91). However, the effect of long-term melatonin therapy with the aim of menstrual cyclicity improvement needs to be evaluated in large-scale prospective randomized studies.

Thanks to the highly selective effects of melatonin on its receptors and therefore an excellent safety profile, it is well tolerated (92). However, more studies are required to be carried out in order to shed light on the possibility of its application in the context of PCOS.

## 4. Discussion

The current review summarizes the role of melatonin in the pathogenesis of PCOS and the protective effects of exogenous melatonin administration in the regulation of reproductive function in the context of PCOS. Melatonin levels in serum and follicular fluid of PCOS patients are different from healthy women. In PCOS patients, melatonin level in serum is usually higher than in healthy women, which is considered as a sign of diagnosing PCOS (32). But a reverse condition occurs in melatonin level of follicular fluid. Due to fewer uptakes of melatonin in ovarian follicle in PCOS patients against healthy women, follicular fluid contains lesser melatonin level compared to the healthy condition (32). Melatonin seems to promote follicular maturation and ovulation through the protection of follicles against oxidative stress and their rescue form atresia (40). Furthermore, melatonin showed protective effects on corpus luteum against ROS via its antioxidant effects (76). Melatonin level in follicular fluid of PCOS women is notably lower than in healthy women, which is related to the ovulation problems. It has been reported that melatonin administration can compensate the reduction of this hormone in follicular fluid and can halt ovulation problems (32). Melatonin treatment indicated protective effects against metabolic and reproductive abnormalities in PCOS patients. Melatonin administration in PCOS patients significantly affects body characteristics including reduced body weight, body mass index and intra-abdominal fat (56). During the ovulatory process, ROS are produced within the follicles; for this reason, the scavenging activity of melatonin plays an important role during ovulation. Melatonin reduces oxidative stress and causes oocyte maturation and luteinization of GC making it as an effective treatment for PCOS patients. Intra-follicular melatonin concentration was considerably lower in PCOS patients giving rise to anovulation and poor oocyte quality in these patients. The administration of melatonin alone or in combination with other drugs in PCOS women has been shown to increase the intrafollicular melatonin concentration, reduce the intrafollicular oxidative stress, and also increases the fertilization and pregnancy rates. Melatonin also improves the production of progesterone from corpus luteum in PCOS patients. The deficiency of melatonin alters gonadotrophin secretion, reduces the synthesis of FSH, and increases the synthesis of LH, the latter being the major change detected in PCOS patients (77). Melatonin can adjust the hypothalamic axis by inhibiting the release of hormones such as FSH, which leads to the reduction of cystic follicles. Melatonin can also regulate the synthesis of GnRH through its receptors in the granulosa-luteal cells via inhibiting GnRH receptor expression and sustaining the corpus luteum which maintains progesterone secretion (93).

## 5. Conclusion

In summary, metabolic dysfunction is the major finding contributing to the initiation of PCOS, melatonin can hinder this process via its improving effects on metabolic functions. Melatonin treatment in PCOS patients can enhance the quality of the oocyte and embryo, increase the number of mature oocytes, reduce obesity, and ameliorate the proinflammatory state, which underlies the development of insulin resistance.

##  Conflict of Interest 

All authors declare no conflicts of interest.
